# Implications of a food system approach for policy agenda-setting design

**DOI:** 10.1016/j.gfs.2020.100451

**Published:** 2021-03

**Authors:** Susanna Kugelberg, Fabio Bartolini, David R. Kanter, Anna Birgitte Milford, Kajsa Pira, Alberto Sanz-Cobena, Adrian Leip

**Affiliations:** a Public Health Consultant, Copenhagen, Denmark; b Department of Agriculture, Food and Environment, University of Pisa, Via del Borghetto 80, Pisa, 56124, Italy; c Department of Environmental Studies, New York University, 285 Mercer Street, 9th floor, New York, NY, 10012, USA; d Norwegian Institute of Bioeconomy Research (NIBIO), Bergen, Norway; e Air Pollution & Climate Secretariat, Första Långgatan 18, Göteborg, Sweden; f Research Center for the Management of Environmental and Agricultural Risks (CEIGRAM), ETSIAAB, Universidad Politécnica de Madrid, Madrid, 28040, Spain; g European Commission, Joint Research Centre (JRC), Institute for Environment and Sustainability, Via E. Fermi, 2749, I-21027, Ispra, VA, Italy

**Keywords:** Agenda-setting, Transformative change, Reflexivity, Directionality, Integrated food policy, Sustainable food system transformation

## Abstract

A call to governments to enact a strategy for a sustainable food system is high on the global agenda. A sustainable food system presupposes a need to go beyond a view of the food system as linear and narrow, to comprehend the food system as dynamic and interlinked, which involves understanding social, economic and ecological outcomes and feedbacks of the system. As such, it should be accompanied by strategic, collaborative, transparent, inclusive, and reflexive agenda-setting process. The concepts of, directionality relating to an agreed vision for a future sustainable food system, and, reflexivity which describes the capacity for critical deliberation and responsiveness, are particularly important. Based on those concepts, this paper proposes an evaluative framework to assess tools and instruments applied during the agenda-setting stage. We apply the evaluative framework to recent food policy processes in Finland and Sweden, revealing that their agenda-setting design cannot be assessed as fully addressing both directionality and reflexivity, thus possibly falling short of the policy design needed for enable more transformative policy approaches.

## Highlights

• Governments need to acknowledge the food system as complex, dynamic and multi-causal.
• Developing a national strategy for a sustainable food system is a powerful leverage that will require more attention to the agenda-setting stage.
• Five policy criteria, based on the concepts of directionality and reflexivity, are defined to assess tools and instruments to enact a strategic, inclusive, and reflexive agenda-setting process.
•Assessment of the Finnish and Swedish food strategy shows that there is scope to improve their designs both on reflexivity and directionality.

## 1 Introduction

The current global food system is largely unsustainable and it is now broadly recognized that a business-as-usual approach is no longer tenable (Rockström et al., 2020; Willett et al., 2019). A shift towards a sustainable food system is a growing subject of interest for policy-makers, recognizing that the food system is an important leverage for a range of issues such as environmental, food and nutrition security, trade, equity and health (European Commission, 2020; IPES-Food (International Panel of Experts on Sustainable Food Systems), 2019). However, despite some positive initiatives, strong responses from governments to enact a national strategy for a sustainable food system have been described as missing, toothless or resulting in only incremental changes rather than meaningful transformation (Candel, 2018; Carey et al., 2015; Marsden et al., 2018). With some exceptions, policies tend to have a singular focus on specific elements of the food system, such as productivity, trade, forestry, fisheries, nutrition or biodiversity (Candel, 2018; Lang and Mason, 2017).

Despite this growing momentum for a sustainable food system, governments face enormous difficulties to shift policy approaches towards sustainability for a number of reasons. Policy-making is influenced by past policies and governance systems, which traditionally favored a productivity agenda (Benton et al., 2019) and can be stuck in institutions and outdated modes of organization that are resistant to change (Hospes and Brons, 2016; Parsons, 2018). Governments often try to integrate sustainability concerns ad-hoc, rather than during ‘upstream’ part of the food policy-process, when decisions are taken on how to spend public money (Galli et al., 2020). In addition, marginalized interests have had limited influence and few opportunities to advocate for food system change (Hospes and Brons, 2016). Moreover, policy visions depends on current political priorities (Meadowcroft, 2011), and not solely on technical evidence and evaluations (Azzam and Levine, 2015). Even where there is widespread political commitment on the need for a sustainable food system, deep disagreement surrounds the day-to-day practical application of what this means in practice (Béné et al., 2019). Just because it is difficult to agree exactly what to prioritize, and to what extent, however, is no reason to rule out efforts to integrate sustainability objectives. The point with a holistic and integrated food system approach is precisely to ensure that responsibility for prevention of environmental degradation and harm to public health is not left to individuals and producers, but is pro-actively embedded in food system activities (Lang and Mason, 2017). The key challenge for governments is to address sustainability concerns —which include environmental sustainability, human health, economic and social — through a *food system approach* (Caron et al., 2018; Recanati et al., 2019; Rockström et al., 2020). A food system approach presupposes a holistic and comprehensive understanding of how the interlinkages between food production, processing, distribution and consumption contributes to a sustainable and healthy diet (Willett et al., 2019). Moreover, it emphasizes the need to go beyond the food system as linear and “single-minded” to comprehend the food system as complex and dynamic, which involves social, economic and ecological outcomes and feedbacks of the system (Ericksen, 2008; Gillespie and van den Bold, 2017; Ingram et al., 2020). As such, it should be accompanied by a strategic, collaborative, transparent, inclusive, and above all reflexive*,* agenda-setting process. Or, a shift to a sustainable food system will certainly require more innovative governance approaches to enable *new* food policy narratives and ways of conceptualizing problems and solutions (Galli et al., 2020; Recanati et al., 2019; Termeer et al., 2017).

In this paper, we seek to identify governance tools and structures that are compatible with a system approach and can aid policy-makers to develop a more holistic and integrated strategy for a sustainable food system. Two concepts, namely “directionality and “reflexivity”, were identified from the literature related to broader system transformations as being particularly relevant (Weber and Rohracher, 2012) (Table 1). Directionality, relating to a set of governing tools and procedures to enable broad agreement on a future direction and effective vision towards a sustainable food system, and reflexivity, which describes governance tools and instruments to engage diverse food system actors to monitor, evaluate, learn and respond. While these concepts of directionality and reflexivity are instrumental to a broader system transformation, the focus here is how they can support a policy construct of the food system as dynamic and accountable for multiple outcomes, e.g. ecological, climate change, health, nutrition security, social and economic, with complex interlinkages and feedbacks.

**Table 1:** Application of concepts.

Concept		Application	Sources that concepts are indirectly drawn from
Sustainable food systems		A sustainable food system enables a sustainable and healthy diet for all, while taking into account environmental, social and economic dimensions	(Caron et al., 2018; Rockström et al., 2020)
Food System approach		A food system approach requires an integrated and holistic analysis of how the dynamics between food production, processing, distribution and consumption affect a sustainable and healthy diet for all. This presupposes a system thinking and recognition of the multi-causality, multi-actor and non-linearity of the food system. The objective is to identify effective points for action within the system to achieve integrated social, economic and ecological outcomes.	(Ericksen, 2008; FAO, 2018a; Ingram, 2011; Meadows, 2009)
Directionality		The capacity of governments to apply tools and instruments to agree on a future direction and effective vision towards a sustainable food system.	Weber and Rohracher (2012)
Reflexivity		The ability of governments to engage all food system actors to deliberate over current values and practices, and a capacity to monitor and evaluate, learn and respond as creatively, efficiently and responsibly as possible. Otherwise described as a system-wide deliberative engagement process.	Weber and Rohracher (2012)
Prior assessments		Prior systematic assessments and studies (scenario-planning, foresight studies, food system assessments) to set a strategic direction of the food system, and to identify major trade-offs between different outcomes (e.g. health, economic, social and environmental)	
Multi-stakeholder evaluations		A combination of both *formal evaluations* performed by state-led actors and *informal evaluations* by diverse non-state actors across society to have active oversight of complex, multi-level problems using local, regional, national and global stakeholders.	(Ostrom, 1990, 2014)
Formal evaluations		Government-led food system monitoring and evaluation, with the general aim to monitor trends and progress of food system outcomes, based on a standardized approach and methodology	Hildén et al. (2014)
Informal evaluations		Evaluation activities performed by civil society, e.g. universities and non-governmental organizations (NGOs). Although often less standardized they are more prone to be critical and generate insights on other unintended, but relevant, side-effects.	(Hildén et al., 2014; Weiss, 1993)

Although policy-making rarely follows a sequential or step-wise approach, the process can be divided into five major stages: agenda-setting, policy formulation, decision-making, policy implementation and policy evaluation (deLeon, 1999; Howlett and Cashore, 2014). This paper focuses on the agenda-setting stage because this is the initial stage when policy-makers are framing the food system and when key negotiations and decisions about which issues need governments’ attention are prioritized.

On the basis of these concepts, we propose an evaluative framework to assess the potential of agenda-setting tools and instruments to support a sustainable food system approach. We then apply this evaluative framework to recent integrated food policies in Sweden and Finland, developed in 2016 and 2017 respectively, to compare the agenda-setting styles of both countries.

## 2 Development of an evaluation framework

### 2.1 Case study selection

Finland and Sweden are regarded by many as forerunners in terms of integrating environmental (Jordan and Lenschow, 2010) and health concerns in all policies (Shankardass et al., 2018), sustainable diets (Gonzalez Fischer and Garnett, 2016), and climate action policies (GermanWatch, 2019). They are also considered as positive examples in relation to transparency and accountability (Transparency International, 2019). The two countries are relatively comparable in terms of food system characteristics (Andersen et al., 2018) and welfare systems (Esping-Andersen, 1990). The food system strategies of both countries are described below, (see following section 2.4 and Table 3).

### 2.2 Research design

To develop our evaluative framework to assess directionality and reflexivity, we conducted a literature review to give a new interpretation of agenda-setting instruments to enhance directionality and reflexivity. The literature review consisted of different streams of research. One part consisted of reviewed literature related to food and health policy to provide a context and background of enabling and constraining factors to develop an integrated food policy. In parallel, we reviewed approaches related to sustainability transitions that use the concepts of directionality and reflexivity in the context of system change. Further review of policy typologies, tools and instruments from Public Administration and Policy Science was undertaken to provide an interpretation of available policy instruments that are at the disposal of government at the agenda-setting stage. The overall purpose of the literature review was to synthesize and provide an interpretation of what directionality and reflexivity mean in agenda-setting instruments and enabling factors to develop a holistic, participatory and integrated food policy. We primarily used Weber and Rochracher's integrated failures framework (Weber and Rohracher, 2012), from where we adapted the concepts on reflexivity and directionality failures to a set of policy criteria to support a policy shift towards a sustainable food system.

### 2.3 Policy thematic analysis

A number of documents related to the agenda-setting stage, were retrieved via the governmental web portal in each country. We searched for information on institutional structure, past food policies, decision papers on future strategic direction of food policy, commissioned studies and research used in the vision-building process and final Green paper. In addition, we looked for submissions and consultation responses of food system actors, as well as evidence on how the engagement process was conducted (Table 2). More specifically, the analysis examined which procedural instruments, governance arrangements and tools inhibit or enable government capacity for directionality and reflexivity. The submission and summary consultation responses are still available on the Swedish government website, however, they were no longer available on the Finnish government website.

**Table 2:** Documents reviewed for the thematic policy analysis.

Types of Documents	Finland	Sweden
Key Policy outputs released by the government.	Government Bill: *Government report on food policy: Food2030 – Finland feeds us and the world* (Government of Finland, 2010b)	Green Paper: *An attractive, innovative and sustainable strategy for a competitive agriculture and horticulture* (State Public Record (SOU 2015:15), 2015b) Government Bill: *A National Food Strategy for Sweden – more jobs and sustainable growth throughout the country* (Government Offices of Sweden, 2017)
Government inquiries, past and contextual food system policies	Government Bill: *Strategy of sustainable development* (Government of Finland, 2006) National program*: On the promotion of food culture* (Ministry of Agriculture and Forestry, 2008) Government Bill: *Promoting sustainable choices in public procurements* (Government of Finland, 2009) Policy proposal: *Tomorrow's Food – National Food Strategy Proposal* (Ministry of Agriculture and Forestry, 2010) Government Bill*: Food Policy* (Government of Finland, 2010a) Government Bill: *Food Chain Action Plan* (Government of Finland, 2011) *Government Bill: Food safety* (Government of Finland, 2013) Government inquiry: *Critical Success Factors of the Finnish Food Chain* (Lehtonen and Irz, 2013)	Government Bill: *Swedish Environmental Objectives* (Government Offices of Sweden, 1998) National program*: Sweden – the New Culinary Nation, National program (2007–2014)* (Ministry of Innovation and Enterprise, 2007) National program*: Strategy for sustainable consumption* (Ministry of Innovation and Enterprise, 2017b) Government Inquiry: *Growth 2030* (The Royal Swedish Academy of Agricilture and Forestry, 2014) Government Inquiry: *Competition and growth opportunities for Swedish agriculture* (State Public Records (SOU 2014:38), 2014) Government Inquiry: *An attractive, innovative and sustainable strategy for a competitive agriculture and horticulture* (State Public Record (SOU 2015:15), 2015b)
Stakeholder consultation process	*Delegation for a Food Policy Committee* (Ministry of Agriculture and forestry, 2013) Stakeholder survey Food2030: *Productivity of primary production represent a major concern* (Ministry of Agriculture and forestry, 2017)	Dir. 2013:20, 2013*:20* (Government Offices of Sweden, 2014) *List of responses to Green paper* (Ministry of Enterprise and Innovation, 2015a) *Summary of responses to Green Paper* (Ministry of Innovation and Enterprise, 2015)

### 2.4 Food policy context

#### 2.4.1 Finland

Until 2015, the strategic goals of the Finnish food policy were split between different programs and Ministries. On the one hand, food policy aimed for a strong expansion of agriculture and food production for both domestic and export markets (Government of Finland, 2010b) and, on the other hand, it aimed to improve sustainability of the food sector and promote organic and local food (Government of Finland, 2009; Ministry of Agriculture and Forestry, 2008).

In 2016, a new vision was set out in *Food 2030* (Ministry of Agricultre and Forestry, 2016). This replaced the previous government bill, *Food for tomorrow* (Government of Finland, 2010b), and tried to reconcile policy objectives related to market growth and a sustainable food chain (Government of Finland, 2017). The key objectives of *Food2030* show an attempt to consistently address a number of food system challenges (see Table 3), including non-communicable diseases (NCDs) and the role of sustainable and healthy diet in their prevention and treatment, as well as promotion of food culture and local food environments.

#### 2.4.2 Sweden

In 2017, the government bill *A National Food Strategy for Sweden – more jobs and sustainable growth throughout the country* (Government Offices of Sweden, 2017) was adopted and replaced the previous food program *Sweden-the New Culinary Nation* (2007–2014) (Ministry of Innovation and Enterprise, 2007).

The new strategy builds on the previous program's aim of increasing Swedish food exports and employment opportunities in the food chain and, while it aims to align food policy with Swedish environmental objectives (Government Offices of Sweden, 1998), there is a strong emphasis on competitive growth of the food chain (Government Offices of Sweden, 2017).

**Table 3:** Framing of food strategies in Sweden and Finland.

Sweden	Finland
*Food system vision in 2030*	
Swedish food chain in 2030 that is globally competitive, innovative, sustainable and attractive to operate within	The best food in the world and, by 2030, Finnish consumers are eating tasty, healthy and safe Finnish food that has been produced sustainably and ethically. Consumers have the ability and possibility to make informed choices
*Key policy objectives*	
i) a competitive food supply chain while achieving the relevant national environmental objectives ii) to generate growth and employment, while contributing to sustainable development	i) the appreciation of food ii) to strengthen Finland's national brand iii) to ensure responsible food production and distribution iv) to improve the sustainability and competitiveness of the food system, v) to achieve climate and environmental targets vi) to develop and support the food sector vii) to strengthen the role of government as a coordinator viii) to promote the availability of food that is tasty, safe, highly nourishing and reasonably priced and ix) to increase collaboration among food system actors.
*Strategic areas for action to achieve vision and objectives*	
i) Rules and regulations to facilitate a competitive food chain ii) Consumers and markets to increase export and contribute to healthy diets iii) Knowledge and innovation to enable adaptive capacity-building and productivity	i) Primary Production ii) Routes for food from field to table iii) Research, advice and training iv) Food culture and appreciation for food v) Food and public health vi) Food security vii) Competitiveness

### 2.5 Evaluative framework on directionality and reflexivity

Drawing on the literature relating to the transition studies (Grin et al., 2010; Loorbach, 2010) and multi-level perspective (Geels, 2010; Jørgensen, 2012), governance (Candel, 2014; Gillespie and van den Bold, 2017; Hospes and Brons, 2016; Termeer et al., 2017) and sustainable food systems (Béné et al., 2019; Caron et al., 2018; Ericksen, 2008; Rockström et al., 2020), we sought to identify criteria that could be of use for policy-makers and applied in the policy process to navigate a shift towards a sustainable food system approach. Common failures that limit the transformative potential of a given system have also been identified (Weber and Rohracher, 2012). This work suggests that two concepts for navigating system transformation, namely “directionality” and “reflexivity”, can be understood as two essential principles of transformative processes (Weber and Rohracher, 2012). While, not being the only relevant transformative failures, we focus on directionality and reflexivity here, since they have relevance for the agenda-setting stage, when policy is constructed, debated and negotiated.

**Fig. 1:**
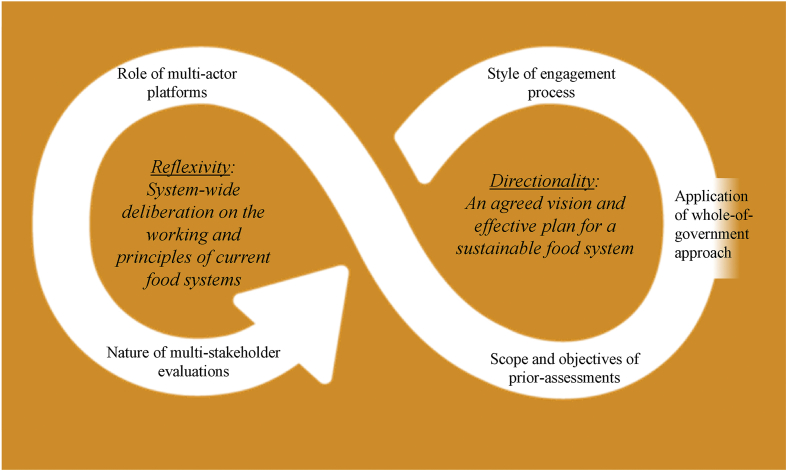
Policy criteria to increase Government Capacity for Reflexivity and Directionality This figure highlights five policy criteria to increase government capacity for directionality and reflexivity to support an integrated food policy, adapted from Weber and Rochracher's integrated failures framework. We use the SDG12 (Sustainable consumption and production) logo as background to emphasize the need of integrating sustainability objectives.

Here we propose five distinct policy criteria to support a sustainable food system approach. Each of these criteria reflect their potential to facilitate meaningful change towards an integrated food systems policy approach — on a four-point scale for each criterion. However, the criteria are *all* equally important and necessary for transformative change, and need to inform a continuous and iterative policy cycle, mirroring the SDG 12 on responsible production and consumption and a holistic and sustainable food system approach (Fig. 1.)

#### 2.5.1 Directionality

A broader food system transformation requires directionality, which means identifying the “grand challenges” (e.g. the SDGs (UN, 2015)) and defining solutions which are agreed by relevant stakeholders (Loorbach, 2010; Weber and Rohracher, 2012). Here we refer to directionality as the capacity of governments to apply tools and instruments to agree on a future direction and effective vision towards a sustainable food system.

The policy design that is required for increasing directionality will therefore need to address policy issues of concerns in a more integrated, collaborative, and inclusive fashion. We propose to assess directionality by looking at the *Scope and objectives of prior asses*sments (policy criterion 1); *Application of whole-of-government approach* (policy criterion 2) and *Style of engagement process* (policy criterion 3).

#### 2.5.2 Reflexivity

However, directionality is not the only precondition to enable a transformative agenda-setting process, transformative processes is also associated with “reflexivity” (Weber and Rohracher, 2012). While reflexivity has multiple and contested meanings (Beck et al., 1994), in the literature related to societal transition the notion of “reflexivity” relates to system's ability to critically reflect on existing values, practices and policies, coupled with their capacity to adapt, (re)design policy instruments and support alternative options (Grin et al., 2010; Sol et al., 2018). The ability of governments to engage all food system actors to deliberate over current values and practices, and a capacity to monitor and evaluate, learn and respond as creatively, efficiently and responsibly as possible. Reflexivity builds on, but goes beyond, directionality (Weber and Rohracher, 2012). It fosters social learning and enables forerunners to understand alternative options and take actions (Sol et al., 2018). Our suggestion is to bring in such reflexive knowledge in the agenda-setting by drawing from *multi-stakeholder evaluations* (policy criteria 4) and connecting it with *multi-actor food policy platforms* (policy criteria 5) to enable a wider set of actors to influence a strategic vision.

#### 2.5.3 Policy criteria on directionality and reflexivity

##### 2.5.3.1 Policy criterion 1: Scope and objectives of prior assessments

To set a future direction for a sustainable food system — and to buffer against ignorance about unintended outcomes—attention is needed to conducting prior assessments and exploratory research to inform the vision-building process. Strategic *prior assessments and studies* include, e.g. impact assessments, research, demonstration projects, foresight studies, and commissioned studies and are examples of agenda-setting instruments Howlett, 2014. However, to ensure that such assessments increase the potential for a shift towards a sustainable food system, they should have wide-ranging scope and objectives and include identification of trade-offs and knowledge gaps (Hebinck et al., 2018; Kanter et al., 2018). For example, one barrier related to agenda-setting of food security policy is that studies tend to focus on only one determinant of future food security, namely quantity of production, often crops (Campbell et al., 2016). This results in less attention to other dimensions, i.e. food availability, access, utilization and stability, in the vision-building process (Vermeulen et al., 2012). Hence, governments need build up capacity during the agenda-setting stage to stimulate debate, provide input and perspectives on relevant food system outcomes, including research to identify trade-offs and knowledge gaps, and clarifying core assumptions.

**Table:**
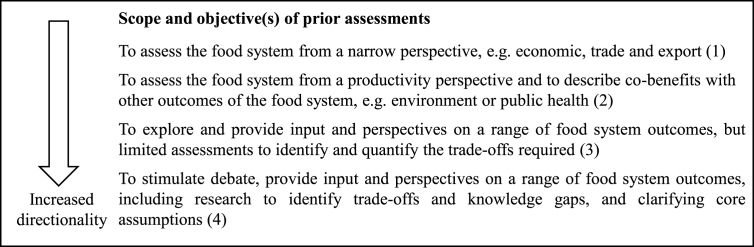
Policy criterion 1 on directionality: Scope and objectives of prior assessments

##### 2.5.3.2 Policy criterion 2: application of whole-of-government approach

Collective coordination is another important part of directionality (Weber and Rohracher, 2012) and requires a conscious effort to engage relevant actors across sectors and levels to agree on strategic priorities. Coordination of food system policies at *governmental* level could be facilitated by formally establishing a “whole-of-government approach” (Kickbusch and Gleicher, 2012) and is a key characteristic of integrated food and health policy (Mikkonen, 2018; Parsons, 2018). The establishment of such joined-up structures at governmental level can take a number of forms, ranging from informal meetings at civil servant level to more formal platforms such as an inter-ministerial committee (Mikkonen, 2018). It has been suggested that such joined-up structures should be formally supported by the highest political authority, e.g. Prime Minister, and function as a collaborative platform to coordinate policy objectives on a long-term basis (FAO, 2018b; Mikkonen, 2018).

**Table:**
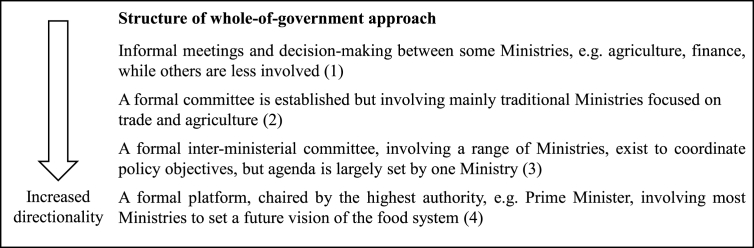
Policy criterion 2 on directionality: application of whole-of-government approach

##### 2.5.3.3 Policy criterion 3: style of engagement process

The transformative potential of food policy development also depends on the extent to which the vision and priorities are *co-designed* and *consensual* with leaders and forerunners representing multiple sectors and at different levels (Totin et al., 2018). In short, transformative projects need to be built on a *shared* vision (Weber and Rohracher, 2012). Success in policy framing is dependent on the extent to which the overall policy framework is agreed upon by key stakeholders who are important for implementation (Howlett, 2012). In policy-making processes, governments are often required by law to consult and engage stakeholders at the agenda-setting stage, e.g. through written stakeholder consultations, parliamentary and public hearings etc. (Howlett, 2014). However, the outcome of the vision-building process depends on *how* the consultation process is conducted. This could vary from being imposed from the top down, where actors are invited to choose between already decided policy options, to being deliberative and collaborative, where actors are able to exert real influence over the decision-making process (Richardson, 2018). It has been argued that a deliberative and collaborative consultation process is more likely to result in a consensual outcome (Richardson, 2018). Thus, to increase directionality, it is important that the engagement process forms an iterative engagement process with all relevant stakeholders involved in jointly deliberating on and setting priorities.

**Table:**
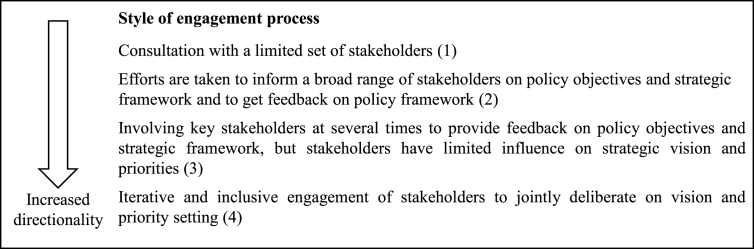
Policy criterion 3 on directionality: style of engagement process

##### 2.5.3.4 Policy criterion 4: nature of multi-stakeholder evaluations

Recognizing that no single actor can possibly oversee the dynamics of change in complex systems, a multi-stakeholder, or polycentric, model of evaluations and active oversight is needed (Ostrom, 2014). This comprises both formal evaluations, performed by state-led actors, and informal evaluations carried out by diverse non-state actors across society. It requires active oversight by local, regional, national and global stakeholders to monitor and solve complex, multi-level problems (Ostrom, 1990, 2014). To maximize their potential to facilitate a shift towards a sustainable food system, formal evaluations should be led by an independent governmental actor and conducted using standardized methodology. Informal evaluations by civil society, e.g. NGOs and universities, are often prone to be more critical (Beck et al., 1994) and are important to highlight new issues or unintended side-effects. For example, civil society evaluations have been important in highlighting the negative consequences of agricultural policies aimed at eradicating hunger on some nutrition outcomes (Fanzo et al., 2013).

**Table:**
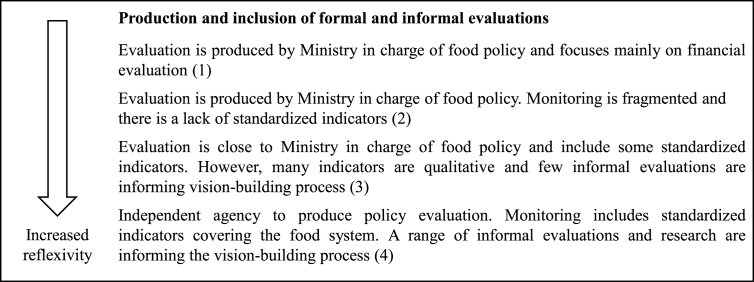
Policy criterion 4 on reflexivity: nature of multi-stakeholder evaluations

##### 2.5.3.5 Policy criterion 5 role of multi-actor food policy platforms

Opportunities or mechanism for reflection and exchange in the form of policy platforms, parliamentary hearings and round-table discussions are important reflexive spaces, where actors can collectively analyse, debate, co-design and support alternative policy options (Bennett and Howlett, 1992; Howlett, 2000). To be most likely to facilitate a reflexive agenda-setting process , the inclusion of opposing and critical views in policy platforms and round-table discussions is important. In addition, processes that allow long-term deliberation are needed, since, as Sabatier notes (Sabatier, 1987), it can take a long time and reflexive processes to bring about changes in value judgements.

**Table:**
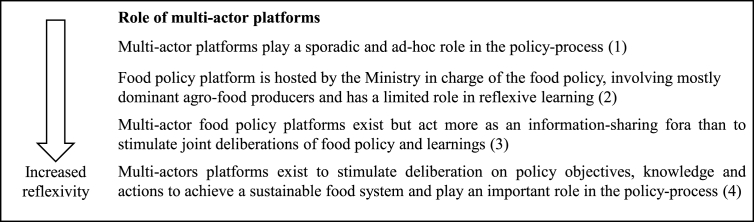
Policy criterion 5 on reflexivity: role of multi-actor food policy platforms

## 3 Application of the proposed evaluative framework to Finland's and Sweden's agenda-setting design

Here we apply the five policy criteria to recent food policy processes in Finland and Sweden.

### 3.1 Application of the evaluative framework to Finland

#### 3.1.1 Scope and objectives of prior assessments

The Finnish vision-building process used a variety of prior assessments and studies, such as research on growth opportunities for the Finnish food chain (Irz et al., 2017), government programs and strategies related to food culture (Ministry of Agriculture and Forestry, 2008) and food tourism (Ministry of Agriculture and Forestry, 2015), the competitiveness of food chain (Government of Finland, 2011), food safety (Government of Finland, 2013), sustainable food procurement (Government of Finland, 2009) and past food policy strategy (Government of Finland, 2010a), as well as food policy developments in other countries, working groups and expert contributions. The key objective of the prior assessments was to explore a wide range of important food system outcomes, such as food culture, food safety, food security, trade, consumption, competitiveness and climate change to guide the future direction of the Finnish food system. Addressing potential trade-offs or incoherencies between policy objectives was largely performed in a descriptive and qualitative way, rather than by quantitative impact assessments. Therefore, we assessed the transformative potential of how the prior assessments were conducted to 3 points.

#### 3.1.2 Application of a whole-of-government approach

Formally the Ministry of Agriculture and Forestry initiated the agenda-setting process of Food2030 and several joined-up mechanisms between ministries were established along the process to align objectives with other political goals related to food and nutrition security and bio-economy (Ministry of Employment and Economy, 2015). To ensure coherence between Food2030's objectives and the government's broader sustainability goals (Government of Finland, 2006), an inter-ministerial group on bio-economy (installed by the previous Government), was specially appointed to deal with this task (Government of Finland, 2015). Currently the Advisory Board for Food Chain, led by the Ministry of Agriculture and Forestry, involves the Ministry of Social Affairs and Health, the Ministry of Education and Culture, the Ministry for Foreign Affairs, the Ministry of the Environment and the Ministry for Finance in food policy-making. Hence, we assessed the transformative potential of Finland's whole-of-government approach to 3 points.

#### 3.1.3 Style of engagement process

The vision was developed through an extensive and iterative engagement process, which started in January 2016 with a large inaugural meeting bringing together over 200 various food system actors to reflect on where Finland wanted to be as a country in 2030, in terms of desirable food system outcomes. The Ministry of Agriculture and Forestry prepared a summary of food policies in other countries and, with the help of an external communication consultancy, organised five workshops between 2016 and 2017, which were attended by over 100 experts representing diverse food system areas. The workshops addressed the profitability and competitiveness of the food system, food security and security of supply, the position of primary production, innovation and a culture of experimentation. In parallel, The Ministry of Agriculture and Forestry also created an online mailbox for submission of feedback and ideas. While the framework of the report was prepared by the Ministry, expert contributions were also requested from parties outside central government. The report was circulated for public comments from 9 September to 28 October 2016. *Food2030* received unanimous support across the political parties when presented to the Parliament in 2016, thus indicating that the engagement process succeeded in building a consensual vision. Thus, we assessed the transformative potential of this highly inclusive and deliberative engagement process to 4 points.

#### 3.1.4 Nature of multi-stakeholder evaluations

Rigorous policy evaluations, both formal and informal, have, with a few exceptions, been scarce e.g. the Sitra Trend List (The Finnish Innovation Fund) which provides an interpretation of the direction of global challenge-related phenomena, known as megatrends (Sitra, 2020). Monitoring of food policy is led by the Ministry of Agriculture and Forestry. The monitoring of indicators in Food2030 covers all relevant food system outcomes but is more descriptive/qualitative than quantitative. Hence, we assessed the nature of multi-stakeholder evaluations to 3 points.

#### 3.1.5 Role of multi-actor food policy platforms

Working across sectors and between ministries — in other words, inter-sectoral action on health — is an established principle in the Finnish policy-making process (Koivusalo, 2010; Puska, 1996). Several multi-actor platforms were active in the agenda-setting process of Food2030 at the national level. A Food Policy Committee was established in 2013, bringing together seven ministries as well as industry, trade and NGOs representatives to coordinate food and nutrition-related policies and to strengthen collaboration within the food chain and between authorities. Currently, the Advisory Board of Food Chain (headed by the Ministry of Agriculture and Forestry) is in place to coordinate implementation across food system actors. Overall, we assessed the broad range of collaborative multi-actor food policy platforms to 4 points.

### 3.2 Application of the evaluative framework to Sweden

#### 3.2.1 Scope and objectives of prior assessments

In the agenda-setting process leading up to the government bill *A National Food Strategy for Sweden – more jobs and sustainable growth throughout the country* (Government Offices of Sweden, 2017), the Swedish government commissioned an expert group to provide a strategic assessment to support decision-making on a vision for Sweden's food system by 2030 (State Public Record (SOU 2015:15); State Public Records (State Public Records (SOU 2014:38), 2014, State Public Record (SOU 2015:15), 2015a); The Royal Swedish Academy of Agricilture and Forestry, 2014). The task of the expert group was to elaborate a strategy for long-term competitive growth of Swedish food production, while addressing challenges related to climate change, the growing international competition of food markets, adaption towards commercial farming and considering how research, innovation and regulatory framework can strengthen food production and facilitate food entrepreneurship (Dir. 2013:20). Investigation of potential synergies with the national innovation strategy (Ministry of Innovation and Enterprise, 2012) and potential socio-economic impact as well as effects on gender and/or other Swedish environmental objectives (Government Offices of Sweden, 1998) was also asked for (Dir. 2013:20). The resulting expert assessments mainly provide an account of opportunities to increase productivity and growth by investigating how it can be stimulated by a simplified regulatory framework, new consumer demands or markets, and creating linkages between food chain actors and innovation and research (State Public Record (SOU 2015:15), 2015b; State Public Records (SOU 2014:38), 2014; The Royal Swedish Academy of Agricilture and Forestry, 2014). In addition, they highlight positive trends in the Swedish agricultural system that fit a sustainability narrative (State Public Record (SOU 2015:15), 2015b; State Public Records (SOU 2014:38), 2014). For example, climate change is described as mostly a positive driver for Swedish food exports, since a warmer climate would increase Swedish productivity and market demand (because it would simultaneously have a negative effect on productivity in the south) (State Public Record (SOU 2015:15), 2015b). Hence, we assessed the range of prior assessments to 2 points.

#### 3.2.2 Application of whole-of-government approach

While the ambition was to set a common direction in which the entire food supply chain works together (Government Offices of Sweden, 2017), the agenda-setting process to develop the Swedish Food policy reflects more a departmentalized approach, initiated and coordinated by the Ministry of Enterprise and Innovation (Government Offices of Sweden, 2014), with no formal joined-up structures in place at the time when the policy was developed to facilitate a whole-of-government approach, e.g. an established inter-ministerial committee. Thus, we assessed the Swedish whole-of-government approach to 2 points.

#### 3.2.3 Style of engagement process

The Swedish consultation process was initiated by the Ministry of Innovation and Enterprise, who invited food system actors to provide their feedback at two stages, after the commissioned expert study and then after the release of the Green paper. While the consultation processes had a broad scope, i.e. it consisted of both written submissions (Ministry of Enterprise and Innovation, 2015b) from diverse food system actors (Ministry of Enterprise and Innovation, 2015a) and “dialogue-meetings” with 700 food system actors, the process did not allow for much stakeholder influence over the strategic priorities, which had already been identified in the commissioned study (Ministry of Enterprise and Innovation, 2016). In addition, the government bill was preceded by long negotiations between the Government and the political parties on the strategic goals and objectives, which suggests that there was some disagreement at the political level about the scope and content of the policy (Ministry of Innovation and Enterprise, 2017a). Thus, we assessed the engagement process to 3 points.

#### 3.2.4 Nature of multi-stakeholder evaluations

The role of multi-stakeholder evaluations in the vision-building process was mainly to assess opportunities to increase productivity and growth (The Royal Swedish Academy of Agricilture and Forestry, 2014). The Ministry of Innovation and Enterprise is responsible for evaluating food policy and specifically in terms of budget allocations (Government Offices of Sweden, 2017). Although there are informal food policy evaluations available in Sweden, strong influence on the vision-building process by evaluations from informal, and often more critical, non-state actors, was not identified. Overall, we assessed transformative potential of the multi-stakeholder evaluations to 2 points.

#### 3.2.5 Role of multi-actor food policy platforms

No specific formal platform was identified, at the time when the policy was drafted, to engage multiple actors in capacity-building and collaborative efforts at the national level to support the implementation of a sustainable food system. Hence, we assessed the role of multi-actor food policy platforms in the vision-building process to 1 point.

**Fig. 2:**
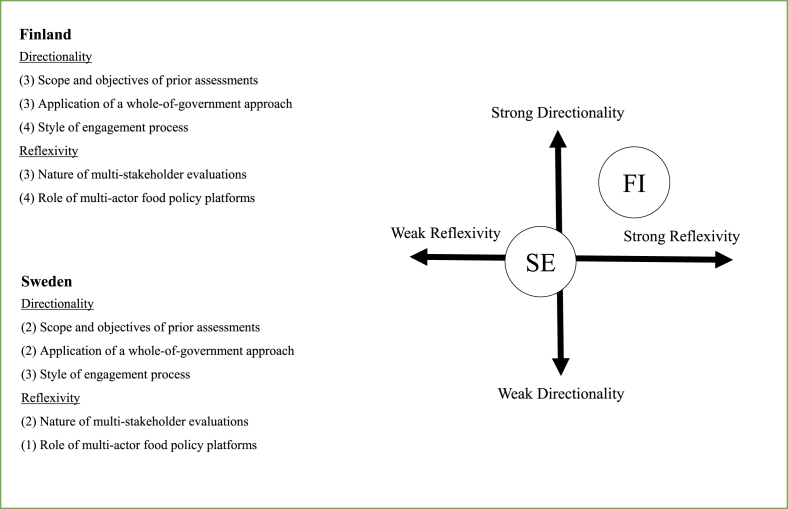
Assessments of agenda-setting design in Finland and Sweden (numbers refer to the score for each of the five assessment criteria) Fig. 2 illustrates the potential of applied policy measures in Finland and Sweden to facilitate a strategic, collaborative, inclusive and reflexive agenda-setting process.

## 4 Discussion

Building on the concepts of directionality and reflexivity, we developed a framework to assess how agenda-setting tools and instruments can contribute to an open, collaborative and reflexive agenda-setting process, and ideally the enactment of a holistic and integrated food system strategy. Each of the criteria in our framework represent an agenda-setting process or instrument, and application of the four-point scale in our framework is designed to support a shift to a more holistic and integrated food system approach.

We applied the framework to the agenda-setting process followed in Sweden and Finland during development of recent food policies. The results show that there are some key differences in policy styles, where Finland — to a higher extent — applies policy criteria that increase the potential for directionality and reflexivity (Table 2). As a result, Finland's food policy is framed in a way that acknowledges several sustainability dimensions as integral to their food policy's vision and objectives, reflecting a holistic approach to the food system.

Sweden applies policy criteria on directionality and reflexivity to a much lesser extent and the framing of Sweden's food policy largely reflects a narrow and linear approach, emphasizing competiveness of the food supply chain, growth and employment.

If a government calls for a long-term food system transformation towards a sustainable food system, then it is necessary to invest in instruments that increase both directionality and reflexivity. Crucially, this means that prior assessments on a range of sustainability dimensions need to be commissioned, formal and informal evaluations conducted on the dynamics of the food system and that these have to be paired with the vision-building process, negotiated across the government and in multi-actor platforms and agreed in a deliberative consultation process with groups of stakeholders. And for each of these processes the four-point scale in our proposed framework could be applied to maximize the overall potential for holistic and systemic solutions.

A distinctive aspect of the Finnish agenda-setting design, for example, is that while tools and instruments are applied to address some aspects of directionality, less is done to address reflexivity. The Finnish food policy is built on a highly participatory and explorative vision-building process, which resulted in an inclusive and holistic food policy. However, an inclusive food policy is not the same as an integrated food policy. An inclusive policy, where all desirable outcomes-from economic to social and environmental-are included but side-tracked, can be described as a “more of everything” policy vision (Lindahl et al., 2017), and has been criticized as being unrealistic and as effectively masking an economic agenda (ibid).

From a transformative perspective towards a sustainable food system, our framework confirms that such a “more of everything” vision-building approach is problematic. Firstly, it undermines the achievement of a truly shared, powerful and strategic future direction of the food system. In other words, it impedes directionality, which also requires robust analysis of potential trade-offs between the different food system outcomes. Secondly, ignorance about potential conflicts largely hinders *reflexive* thinking about current and detrimental practices. It may also undermine the perceived legitimacy of selected policy priorities and interventions, if these have not been reached on the basis of a fully informative and agreed process (Carbone, 2008).

A major hindrance to building capacity for reflexivity, is that, while informal policy evaluations from non-governmental organizations are often ignored or suffer from unorthodox methodologies making it difficult to draw conclusions, formal policy evaluations tend to be close to the lead Ministry involved and, therefore, are often less critical (Hildén et al., 2014). Even when data collection and indicators are standardized, the monitoring frameworks often lack standardized indicators covering the whole food system (Kanter et al., 2018).

Our evaluative framework, based on the concepts of directionality and reflexivity, adds to a growing academic literature that claims that a shift towards a sustainable food system is a collective, multi-level and multi-scale endeavor that requires a governance framework and policy-process that is designed to enable systemic solutions (Béné et al., 2019; Caron et al., 2018; Galli et al., 2020; Rockström et al., 2020). Our policy criteria are in no way presented as an exhaustive or exclusive list. Rather, this is a call for further contributions through qualitative and investigative research, with the goal of identifying further factors that can increase governments' capacity for navigating food system transformation towards a sustainable food system. While this will involve assessing countries’ food policy implementation performance (FAO, 2018b), it also puts more emphasis on fine-tuning the instruments and procedures available to governments at the agenda-setting stage to lay the foundations for governing food system transformation based on a system thinking.

Recognizing that food systems contain very complex relationships, we limited the analysis to the elements that have theoretical relevance for our framework. Hence, we focus on the agenda-setting stage of policy development, where food policy narratives are being debated and constructed. Thus, we have not analyzed the implementation framework and its implications for a strategy for a sustainable food system. When assessing food system strategies from Finland and Sweden we have mostly relied on publicly available documents found on the governments’ web portals. However, we recognize that interviews, focus groups and wider stakeholder workshops could provide us with valuable feedback on policy indicators and further insights on other policy instruments to enable an integrated food policy approach. We found that directionality and reflexivity are not always easily translated to concrete and practical implications for policy-making, since these concepts will interplay with evaluative judgements about how the food system is conceptualised. Hence, our view of the food system as a multi-actor, non-linear, multi-causal and dynamic system has influenced choices and scale of criteria.

## 5 Conclusion

Developing a shared policy vision towards a future sustainable food system is a powerful, collective excersize that needs to be accompanied by a strategic, transparent, inclusive, and reflexive agenda-setting process to reach its full transformative potential. To support governments in their decisions to enact a new policy agenda on a sustainable food system, we propose an evaluative framework based on the concepts of directionality and reflexivity, to assess the available policy toolbox at the agenda-setting stage to set the stage for a holistic and integrated food policy approach.

Illustration of this framework to the Finnish and Swedish food system strategies reveals that for both countries - though generally regarded as forerunners in integrating environmental and health concerns in all policies - their agenda-setting design cannot be assessed as fully addressing both directionality and reflexivity, thus possibly falling short of the policy design needed for enable more transformative policy agendas. This confirms the need to reform rigid policy making processes and the importance of establishing robust mechanisms and processes that include all stakeholders — across and beyond government — and give a voice to those that are often marginalized from such processes, as well as conducting broad-ranging prior assessments and rigorous formal and informal evaluations.

## Declaration of competing interest

All the authors declare no Conflict of Interest to the Manuscript:
